# Supplementary material for: Fast, multiplexable and efficient somatic gene deletions in adult mouse skeletal muscle fibers using AAV-CRISPR/Cas9
Source: Nat Commun. 2023 Sep 30;14:6116. doi: 10.1038/s41467-023-41769-7 (PMC10542775; doi:10.1038/s41467-023-41769-7)
Supplement: Supplementary file 1 — Supplementary Information [file 41467_2023_41769_MOESM1_ESM.pdf]

Supplementary Materials for

Fast, multiplexable and efficient somatic gene deletions in adult mouse skeletal muscle fibers using AAV-CRISPR/Cas9

Marco Thürkauf *et al.*

*Corresponding author: Markus A. Rüegg, email: mailto:markus-a.ruegg@unibas.ch

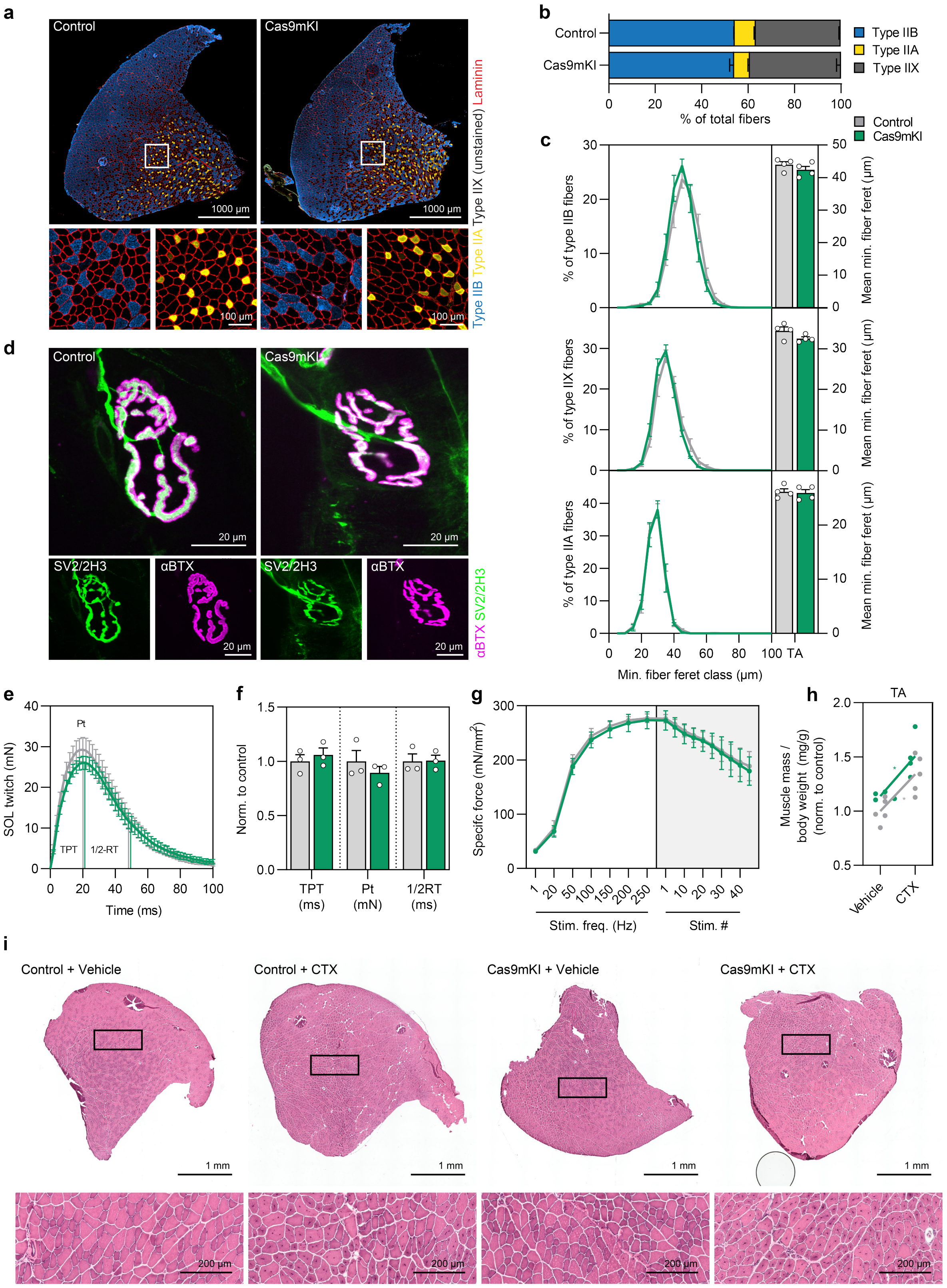

a
Control
Cas9mKI
1000 µm
100 µm
Type IIB Type IIA Type IIX (unstained) Laminin
b
Control
Cas9mKI
Type IIB
Type IIA
Type IIX
0 20 40 60 80 100
% of total fibers
c
Control
Cas9mKI
% of type IIB fibers
% of type IIX fibers
% of type IIA fibers
Mean min. fiber feret (µm)
Min. fiber feret class (µm)
TA
d
Control
Cas9mKI
20 µm
SV2/2H3
αBTX
αBTX SV2/2H3
e
Pt
TPT
1/2-RT
SOL twitch (mN)
Time (ms)
f
Norm. to control
TPT (ms)
Pt (mN)
1/2RT (ms)
g
Specifc force (mN/mm²)
Stim. freq. (Hz)
Stim. #
h
TA
Muscle mass / body weight (mg/g) (norm. to control)
Vehicle
CTX
i
Control + Vehicle
Control + CTX
Cas9mKI + Vehicle
Cas9mKI + CTX
1 mm
200 µm

Figure S1: **Expression of Cas9 in skeletal muscle fibers does not affect muscle physiology. a** Cross sections of *tibialis anterior* (TA) muscle stained for type IIB (blue), type IIA (yellow) fibers and laminin (red) from control and Cas9mKI mice. **b** Quantification of the TA fiber-type composition. **c** Minimal fiber feret distribution (left) and mean minimal fiber feret (right) of TA muscle of control (grey) and Cas9mKI (green) mice according to their fiber type. **d** Whole-mount view of neuromuscular junctions of the *extensor digitorum longus* (EDL) muscle stained for the motor nerve using a mixture of antibodies directed against synaptic vesicle glycoprotein 2A (SV2; green) and neurofilament (2H3; green), and for postsynaptic AChRs with α-bungarotoxin (αBTX; magenta). **e** Ex-vivo twitch response of isolated *soleus* (SOL) muscle from Cas9mKI and control mice. Peak twitch (Pt), time-to-peak twitch (TPT) and half-relaxation time (1/2RT) are indicated. **f** Quantification of ex-vivo twitch response parameters (TPT, Pt, 1/2RT) of isolated SOL muscle from Cas9mKI and control mice. **g** Force-frequency curve (left) and fatigue response to multiple stimulations (right) of SOL muscle from control and Cas9mKI mice. **h** Changes in muscle mass of TA muscle of control or Cas9mKI mice 21 days post cardiotoxin (CTX) injury. While there is no significant difference between control and Cas9mKI mice, muscles are significantly heavier after regeneration ($P < 0.05$). **i** Cross sections of TA, stained for hematoxylin and eosin, of uninjured (vehicle) and injured (CTX) from control and Cas9mKI mice, 21 days post injection. Data are means ± SEM. For **b** and **c**, n = 4 mice. For **e – g**, n = 3 mice. For **h**, n = 5 (control) and 4 (Cas9mKI) mice. None of the data in **b, c, e – g** are not significantly different between control and Cas9mKI mice ($P > 0.05$) using unpaired student's two-sided t-test. For **h**, statistical significance is based on two-way ANOVA with Tukey's post-hoc test. *$P < 0.05$. Source data and precise p-values are provided as a source data file.

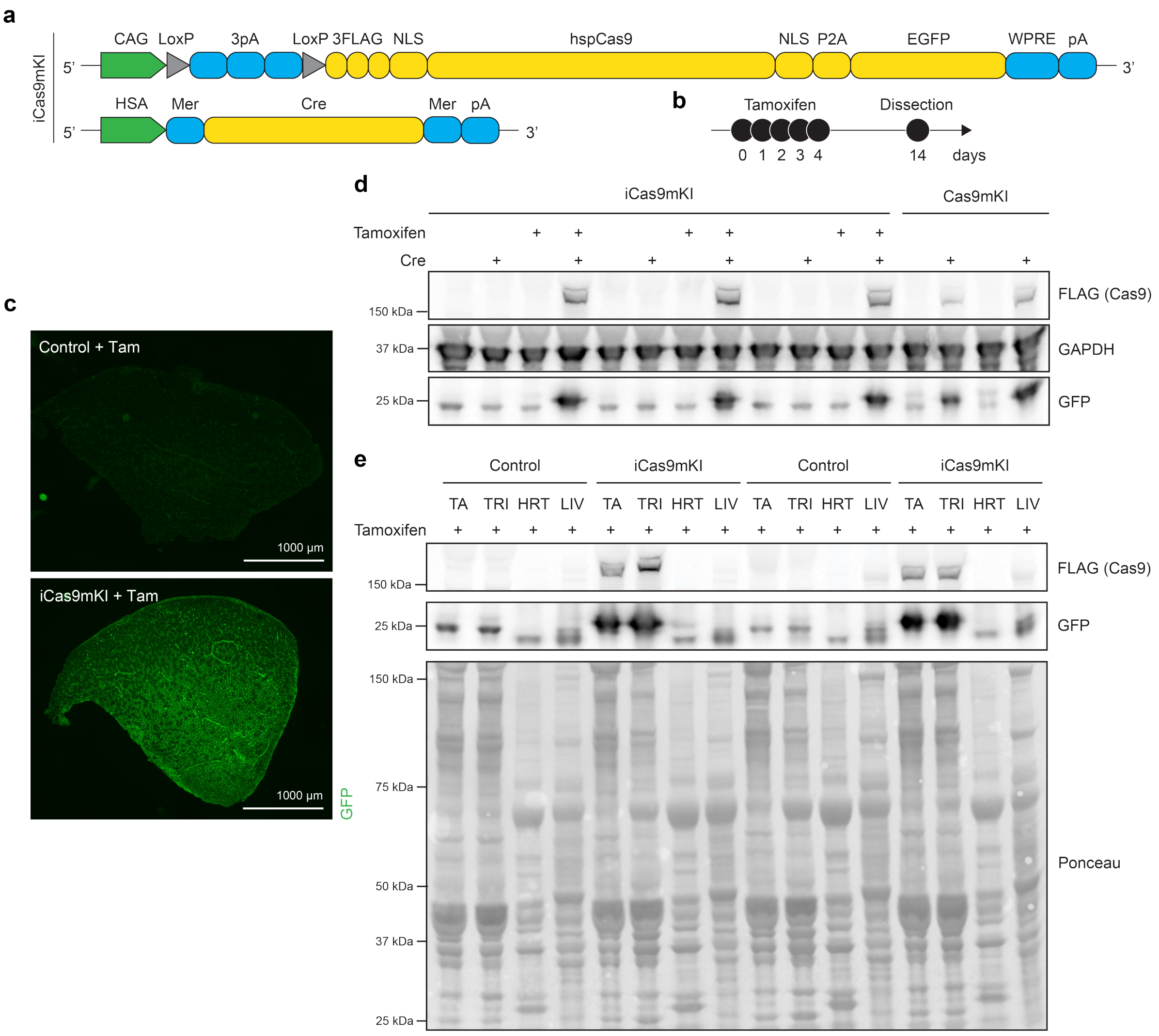

Figure S2: **Validation of iCas9mKI mice. a** Schematic of the iCas9mKI mouse model. For abbreviations, see legend to Figure 1. Mer: mutated estrogen receptor. **b** Timeline of tamoxifen injection and mouse analysis 14 days post-injection. **c** Cross sections from *tibialis anterior* (TA) muscle from control and iCas9mKI mice stained for GFP. **d** Western blot analysis of TA muscle for the FLAG-tag and GFP in Cre-positive or Cre-negative iCas9mKI and Cas9mKI mice upon tamoxifen injection. Glyceraldehyde-3-phosphate dehydrogenase (GAPDH) was used as loading control. **e** Western blot analysis for the FLAG-tag and GFP in TA, *triceps brachii* (TRI), heart (HRT) and liver (LIV) after tamoxifen administration in control and iCas9mKI mice. Ponceau staining of the transferred proteins was used as loading control. Source data are provided as a source data file.

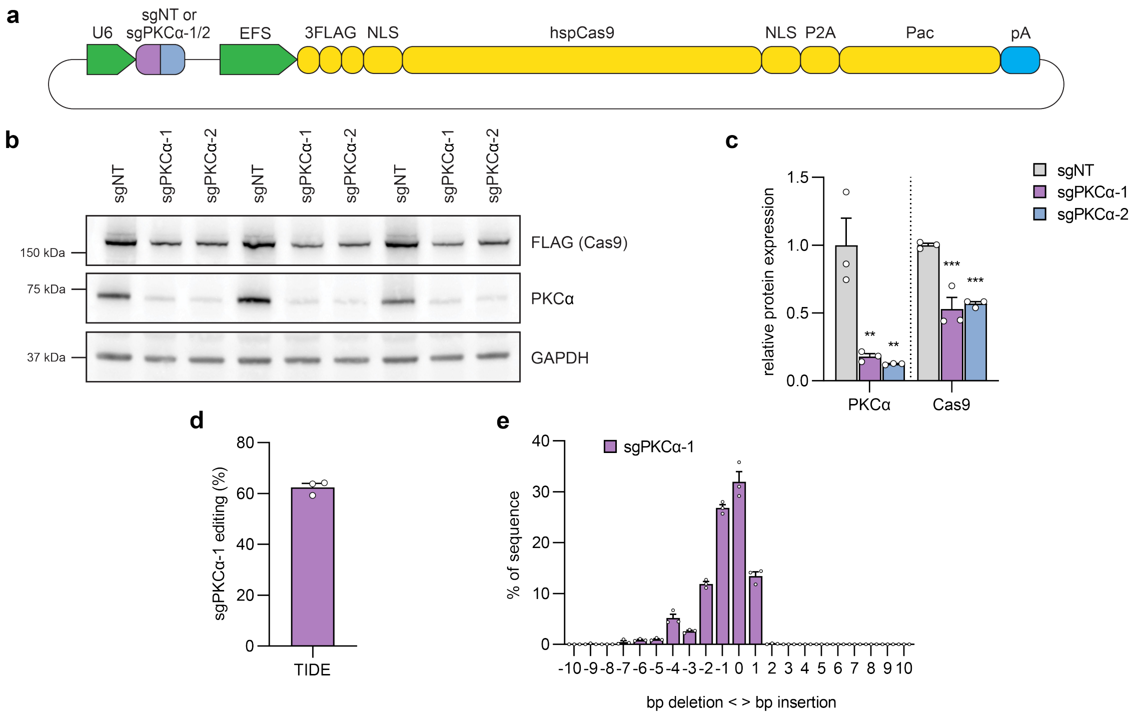

Figure S3: **Efficiency testing of sgRNAs in cultured C2C12 myotubes. a** Schematic of the expression plasmid used for transfection of C2C12 myoblasts. For abbreviations, see legend to Figure 1. U6: human U6 promoter; EFS: eukaryotic translation elongation factor 1 α short promoter; Pac: puromycin N-acetyltransferase. **b** Western blot analysis for FLAG-tagged Cas9 and PKCα using lysates from C2C12 myotubes after transfection with the indicated constructs and puromycin selection. **c** Quantification of protein abundance. **d** Tracking of Indels by Decomposition (TIDE) analysis of Cas9/sgPKCα-1-expressing cells. **e** Frequency distribution of the DNA editing events around the binding site of sgPKCα-1. Data are means ± SEM with n = 3 wells for each sgRNA. Significance was determined using one-way ANOVA with Fishers LSD post-hoc test. *P < 0.05, **P< 0.01, ***P < 0.001. Source data and precise p-values are provided as a source data file.

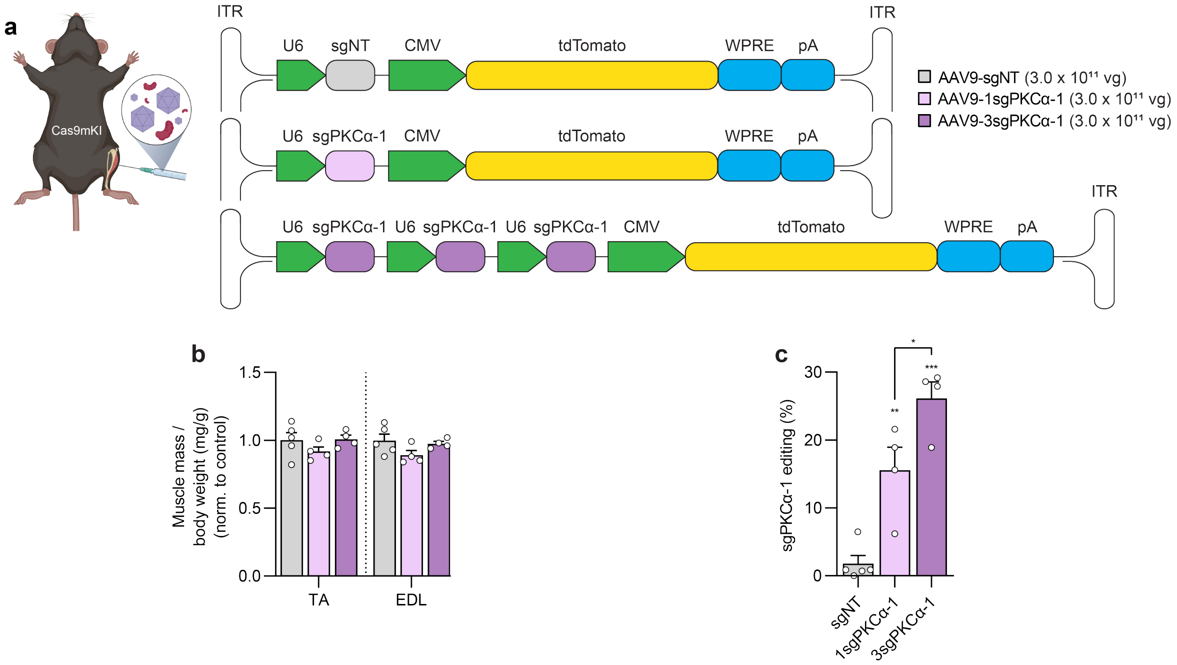

Figure S4: ***In vivo* efficacy testing of one versus three sgPKCα-1 expression cassettes.** **a** Schematic illustration of experimental procedure and targeting construct. For abbreviations, see legend to Figure 2. **b** Relative mass of *tibialis anterior* (TA) and *extensor digitorum longus* (EDL) muscle of AAV9-sgNT (grey), AAV9-1sgPKC α-1 (light purple) or AAV9-3sgPKCα-1 (purple) injected Cas9mKI mice. **c** TIDE analysis of TA muscle, injected with AAV9-sgNT, AAV9-1sgPKCα-1 or AAV9-3sgPKCα-1. Data are means ± SEM. n = 5 (AAV9-sgNT) and 4 (AAV9-1sgPKCα-1, AAV9-3sgPKCα-1) mice. Statistical significance is based on one-way ANOVA with Fishers LSD post-hoc test. *P < 0.05, **P < 0.01, ***P < 0.001. Experimental scheme in **a** was created with BioRender.com. Source data and precise p-values are provided as a source data file.

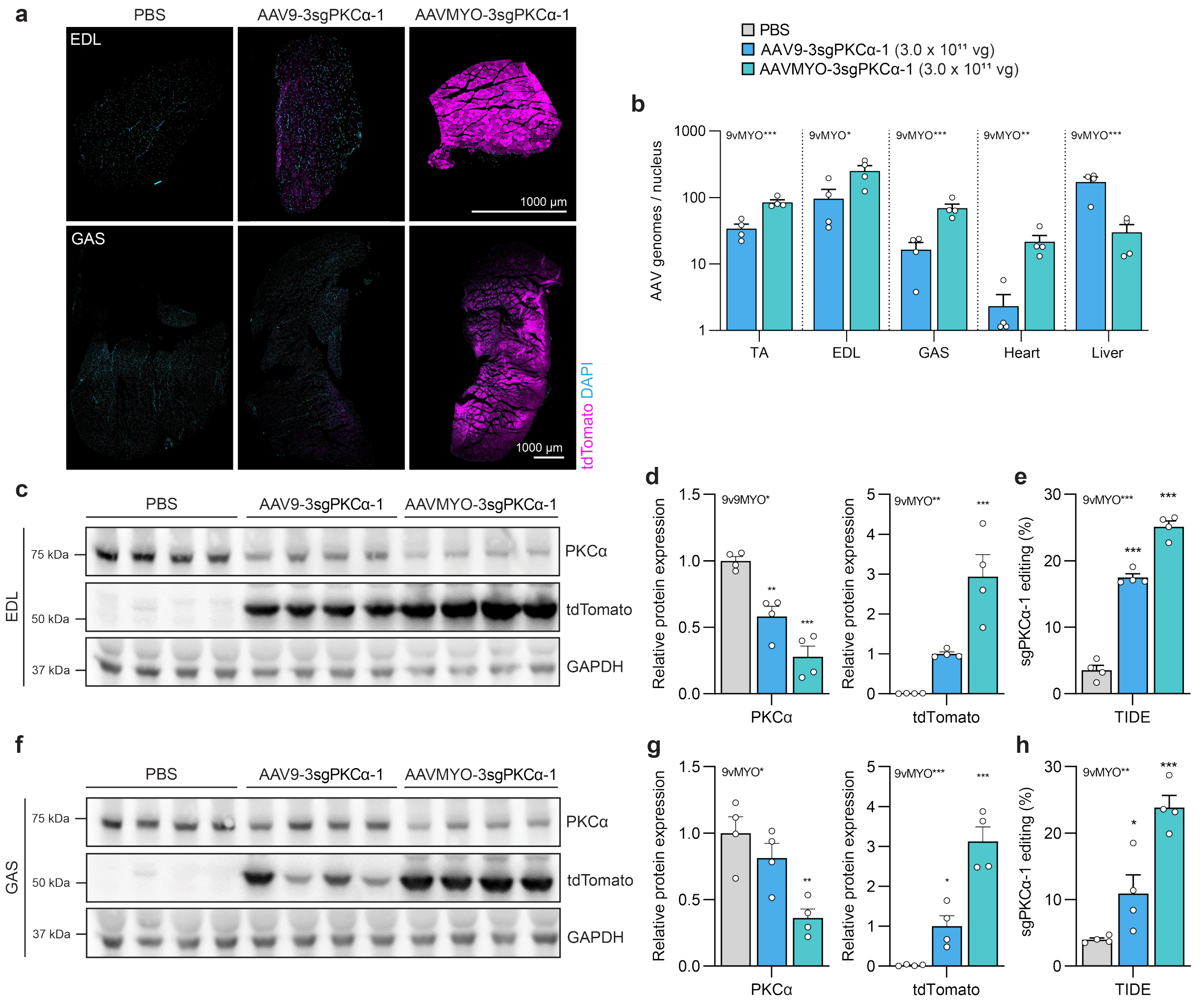

Figure S5: **AVVMYO-mediated sgRNA delivery into TA muscle induces a strong reduction of PKCα in nearby muscles. a** Representative images of cross sections of *extensor digitorum longus* (EDL) and *gastrocnemius* (GAS) muscle stained for tdTomato (magenta) and DAPI (blue). **b** Distribution of AAVs in TA, EDL, GAS, heart and liver upon intramuscular injection of AAV9-3sgPKCα-1 (light blue) or AAVMYO-3sgPKCα-1 (cyan) into Cas9mKI mice. **c, f** Western blot analysis for PKCα and tdTomato of the indicated muscles and conditions. **d, g** Quantification of Western blots shown in c and f for the indicated proteins. Results for PKCα were normalized to the levels in PBS-injected muscles (grey). For tdTomato, levels of AAV9-3sgPKCα-1-injected muscle were set to 1. **e, h** TIDE analysis for sgPKCα-1 at its target site. Data are means ± SEM. n = 4 mice. Significance was determined using one-way ANOVA with Fishers LSD post-hoc test (**d,**

e, g, h) or unpaired student's two-sided t-test (**b**). *P < 0.05, **P < 0.01, ***P < 0.001. Source data and precise p-values are provided as a source data file.

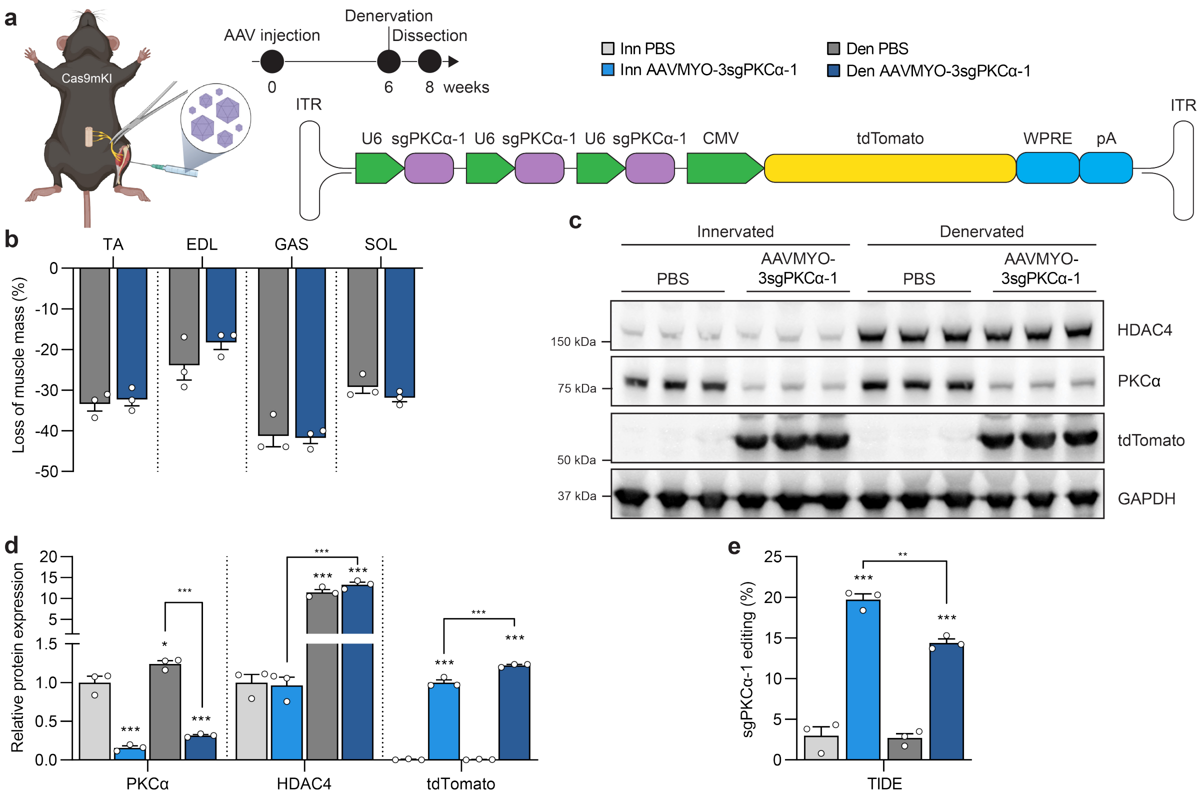

Figure S6: **AAVMYO-CRISPR/Cas9-induced depletion of PKCα in denervated muscle. a** Schematic presentation of the experimental procedure. **b** Loss of mass after 14 days of denervation of *tibialis anterior* (TA), *extensor digitorum longus* (EDL), *gastrocnemius* (GAS) and *soleus* (SOL) muscles after normalization to the innervated, contralateral muscle in each animal. **c** Western blot analysis and **d** quantification of protein abundance of PKCα, HDAC4 and tdTomato in either innervated (light colors) or denervated (dark colors) TA muscle of Cas9mKI mice injected with PBS (grey colors) or AAVMYO-3sgPKCα-1 (blue colors). **e** TIDE analysis of the sgPKCα-1-targeted *Prkca* locus in the different conditions. Data are means ± SEM. n = 3 mice. Significance was determined using unpaired student's two-sided t-test (**b**) or two-way ANOVA with Tukey's post-hoc test (**d, e**). *P < 0.05, **P < 0.01, ***P < 0.001. Experimental scheme in **a** was created with BioRender.com. Source data and precise p-values are provided as a source data file.

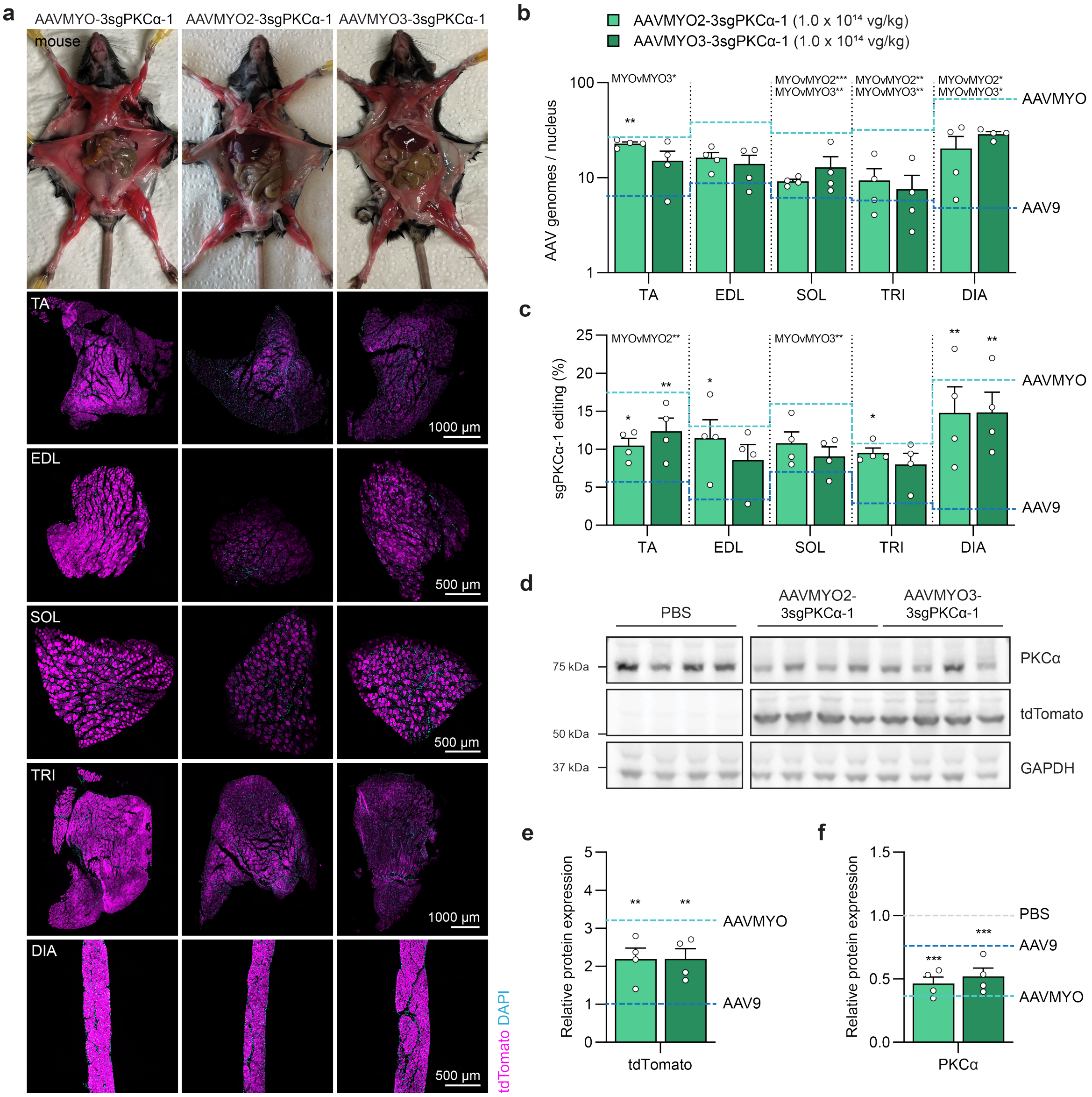

Figure S7: **Efficiency of sgRNA delivery using the liver-detargeted AAVMYO2 and AAVMYO3 compared to AAVMYO. a** Representative images of dissected mice and cross sections of *tibialis anterior* (TA), *extensor digitorum longus* (EDL), *soleus* (SOL), *triceps brachii* (TRI) or diaphragm (DIA) muscle stained for tdTomato (magenta) and DAPI (blue), 6 weeks post-intravenous injection of PBS or AAV (1.0 x 10^{14} vg/kg) into Cas9mKI mice. For comparison, images of AAVMYO- 3sgPKCα-1-injected mice are included, same as on Fig. 4b. **b** Distribution of AAVs in TA, EDL, SOL, TRI and DIA upon intravenous injection of AAVMYO2-3sgPKCα-1 (light green) or AAVMYO3-3sgPKCα-1 (dark green) into Cas9mKI mice. **c** TIDE analysis of the sgPKCα-1-targeted *Prkca* locus.

d Western blot analysis and its quantification (**e, f**) for PKCα (**e**) and tdTomato (**f**) in TA muscle of Cas9mKI mice injected with AAVMYO2 or AAVMYO3, normalized to AAV9-3sgPKCα-1 or PBS respectively. Dashed lines in **b, c, e** and **f** show mean values for AAV9 (light blue), AAVMYO (cyan) and PBS (grey) when appropriate. Samples were loaded on two gels but processed in parallel. Data are means ± SEM. n = 4 mice. Significance was determined using one-way ANOVA with Tukey's post-hoc test. Indication of significance above bars compares to AAV9 (**b, c, e**) or PBS (**f**). *P < 0.05, **P < 0.01, ***P < 0.001. Source data and precise p-values are provided as a source data file.

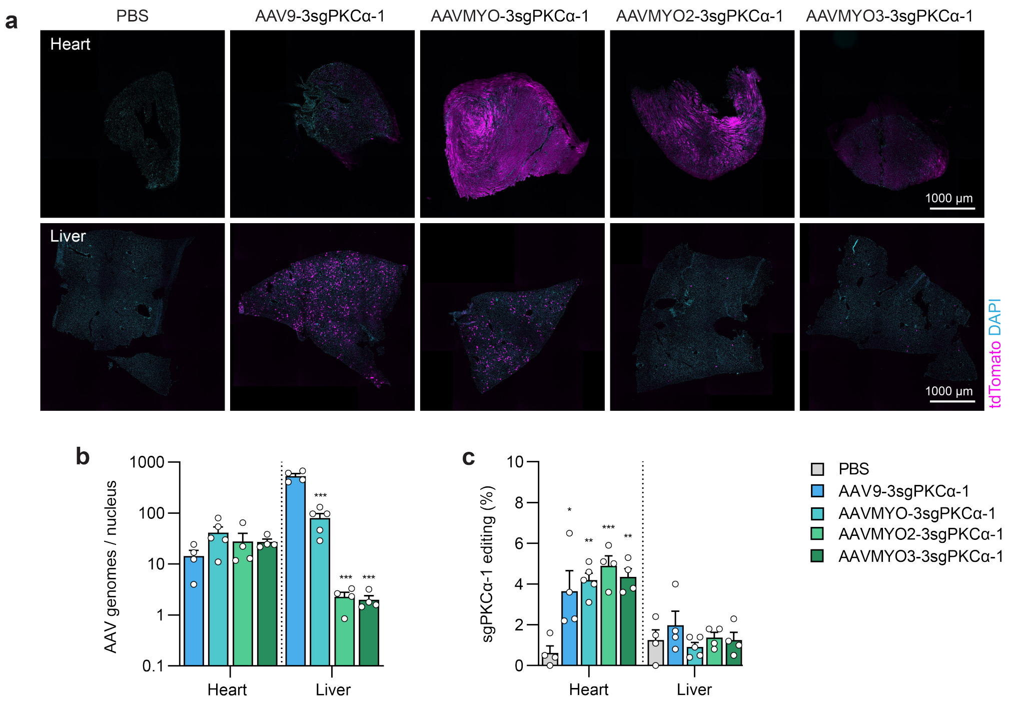

Figure S8: **AAV transduction and DNA editing of heart and liver upon intravenous administration. a** Representative images of heart and liver cross-sections stained for tdTomato (magenta) and DAPI (blue), 6 weeks post-intravenous injection of PBS or AAV (1.0 x 10^{14} vg/kg) into Cas9mKI mice. **b** Distribution of AAVs in heart and liver of Cas9mKI mice upon intravenous injection of PBS (grey), AAV9-3sgPKCα-1 (light blue), AAVMYO-3sgPKCα-1 (cyan), AAVMYO2-3sgPKCα-1 (light green) or AAVMYO3-3sgPKCα-1 (dark green). **c** TIDE analysis of the sgPKCα-1-targeted *Prkca* locus in heart and liver. Data are means ± SEM. n = 4 (Control, AAV9, AAVMYO2, AAVMYO3) and 5 (AAVMYO) mice. Statistical significance is based on one-way ANOVA with Tukey's post-hoc test. *P < 0.05, **P < 0.01, ***P < 0.001. Source data and precise p-values are provided as a source data file.

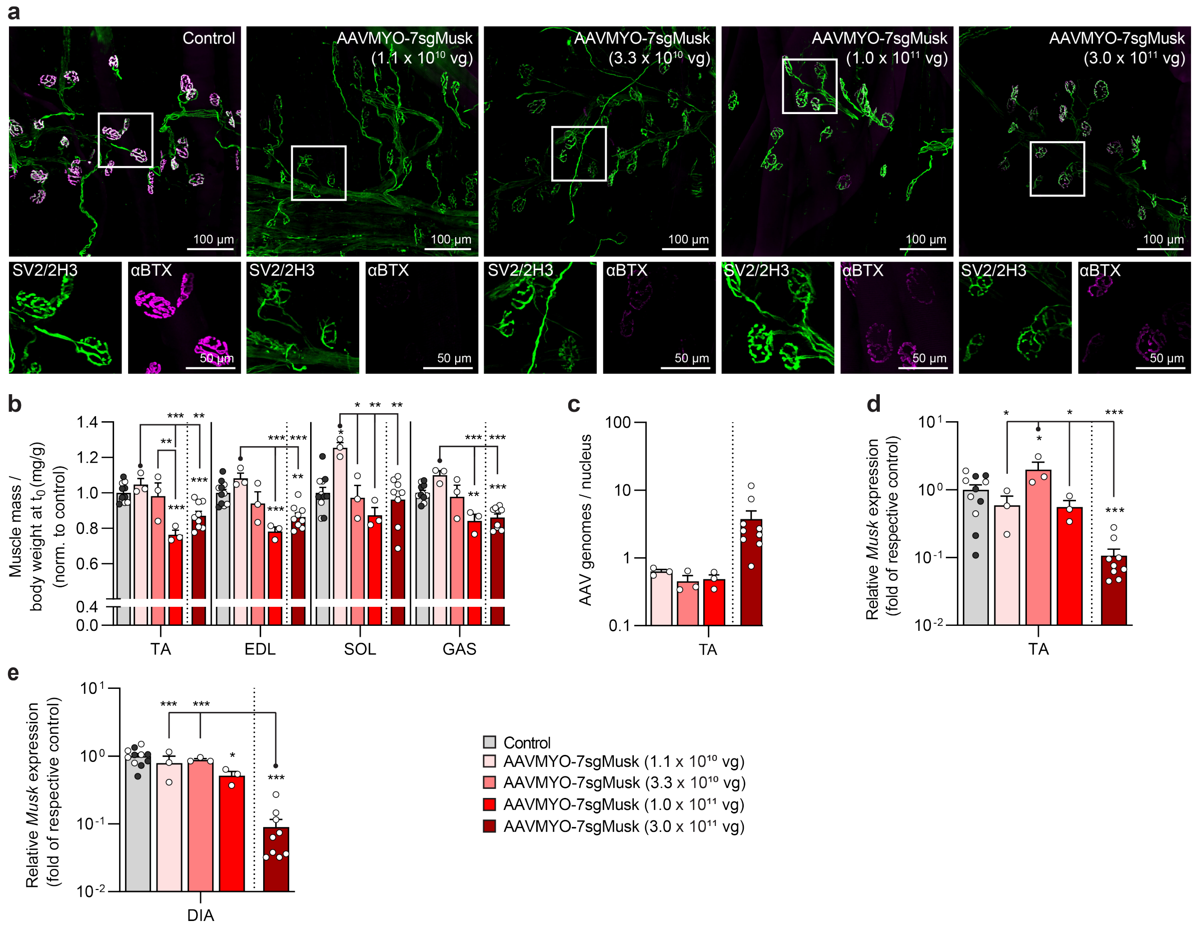

Figure S9: **Intramuscularly injected AAVMYO-7sgMusk spreads to other muscles at high doses to induce a systemic loss of MuSK. a** Representative whole-mount images of *extensor digitorum longus* (EDL) muscles of controls and Cas9mKI mice injected with the indicated amount of AAVMYO-7sgMusk. Presynaptic nerve terminals are stained with a mixture of antibodies directed against synaptic vesicle glycoprotein 2A (SV2; green) and neurofilament (2H3; green). Fluorescently-labeled α-bungarotoxin (αBTX; magenta) was used to visualize postsynaptic AChRs. **b** Changes in mass of the indicated muscles from the contralateral, non-injected leg. Values are normalized to control mice (grey bar; white dots indicate PBS-injected Cas9mKI mice; black dots AAVMYO-7sgMusk-injected control mice). **c** Distribution of AAV in contralateral *tibialis anterior* (TA) muscle of Cas9mKI mice. **d, e** Relative mRNA expression of *Musk* in contralateral TA muscle (**d**) and diaphragm (**e**) of control and AAVMYO-7sgMusk-injected Cas9mKI mice. Data are means ± SEM. n = 11 (Control (5 PBS, 6 AAVMYO)), 3 (1.1 x 10^{10} vg, 3.3 x 10^{10} vg, 1.0 x 10^{11} vg) and 9 (3.0 x 10^{11} vg) mice. Statistical significance is based on one-way ANOVA

with Tukey's post-hoc test. *P < 0.05, **P < 0.01, ***P < 0.001. Source data and precise p-values are provided as a source data file.

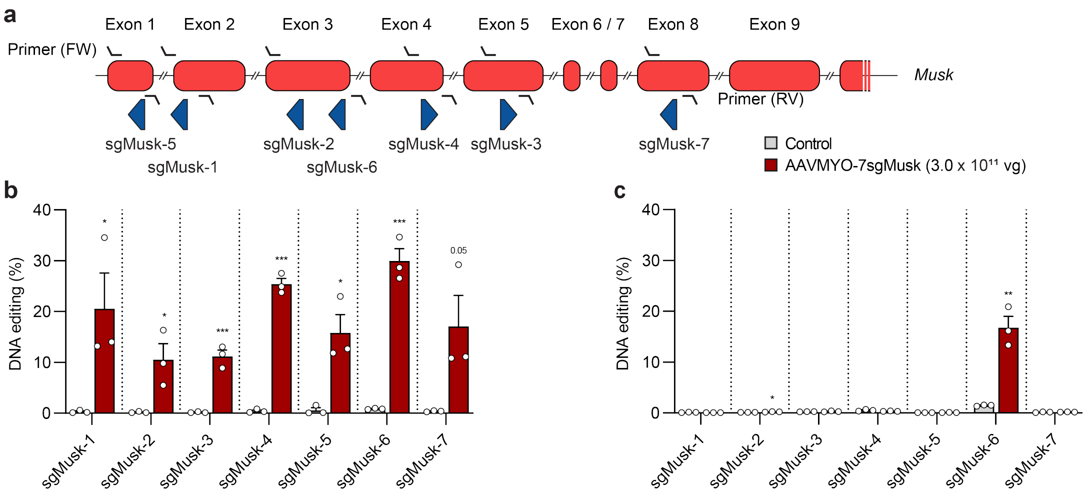

Figure S10: **On/off-target editing by individual sgMusk-(1-7). a** Schematic representation of amplicon design for each sgMusk-(1-7). Location and orientation of each sgRNA, and forward (FW) and reverse (RV) primers for each amplicon are indicated. **b, c** CRISPResso2 analysis for each sgRNA at its *Musk* target site (**b**) and its primary off-target site (**c**). DNA was isolated from *tibialis anterior* (TA) muscle from PBS- (control; grey) or AAVMYO-7sgMusk-injected Cas9mKI mice (red colors). Data are means ± SEM. n = 3 mice. Statistical significance is based on unpaired student's two-sided t-test comparing to control. *P < 0.05, **P < 0.01, ***P < 0.001. Source data and precise p-values are provided as a source data file.

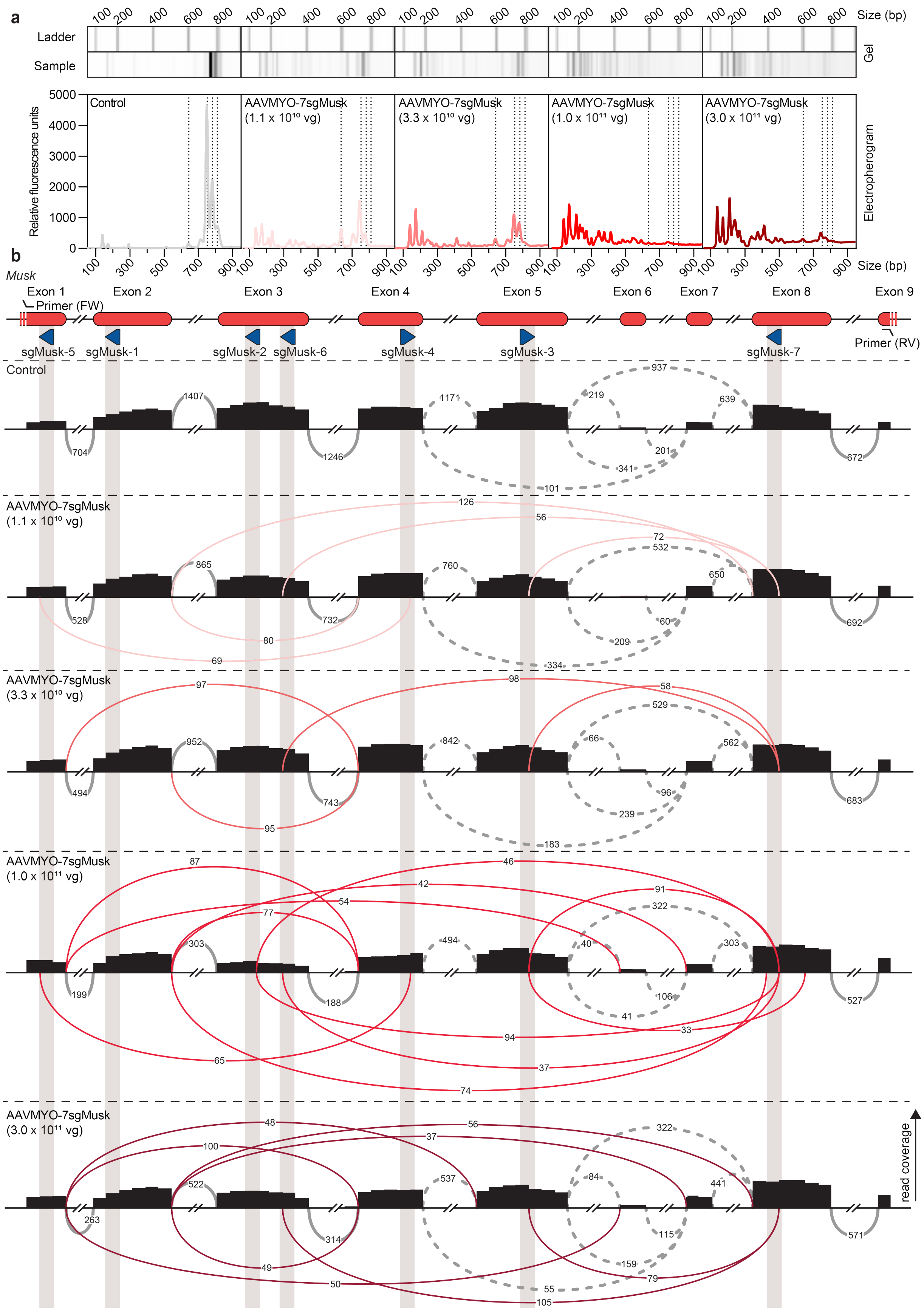
a
Ladder
Sample
Size (bp)
Gel
Relative fluorescence units
Control
AAVMYO-7sgMusk (1.1 x 10¹⁰ vg)
AAVMYO-7sgMusk (3.3 x 10¹⁰ vg)
AAVMYO-7sgMusk (1.0 x 10¹¹ vg)
AAVMYO-7sgMusk (3.0 x 10¹¹ vg)
Electropherogram
b
Musk
Exon 1
Exon 2
Exon 3
Exon 4
Exon 5
Exon 6
Exon 7
Exon 8
Exon 9
Primer (FW)
Primer (RV)
sgMusk-5
sgMusk-1
sgMusk-2
sgMusk-6
sgMusk-4
sgMusk-3
sgMusk-7
Control
AAVMYO-7sgMusk (1.1 x 10¹⁰ vg)
AAVMYO-7sgMusk (3.3 x 10¹⁰ vg)
AAVMYO-7sgMusk (1.0 x 10¹¹ vg)
AAVMYO-7sgMusk (3.0 x 10¹¹ vg)
read coverage

157

Figure S11: **Analysis of *Musk* transcripts upon AAVMYO-7sgMusk injection. a** Fragment analyzer gel (top) and electropherogram (bottom) of PCR fragments for *Musk* cDNA of AAVMYO-7sgMusk-injected (red colors) *tibialis anterior* (TA) muscle of Cas9mKI mice and controls (grey). Primers used for PCR amplification are indicated. Dotted lines indicate size of major PCR products from controls (815 bp: all exons included; 785 bp: skip exon 6; 754 bp: skip exon 6 and 7; 642 bp: skip of exon 5 and 6). **b** Sashimi plot indicating NGS read junctions in control or AAVMYO-7sgMusk-injected Cas9mKI mice. Read junctions observed in control mice are indicated in grey; dotted grey lines are reads from alternatively *spliced* Musk transcripts. Reads junctions occurring only in AAVMYO-7sgMusk-injected mice are indicated in red colors corresponding to the amount of virus used. Height of black squares and numbers represents number of reads of this particular variant. Localization of each sgMusk is highlighted in brown. Exons are drawn to scale. Data represents 1 mouse per condition. Source data are provided as a source data file.

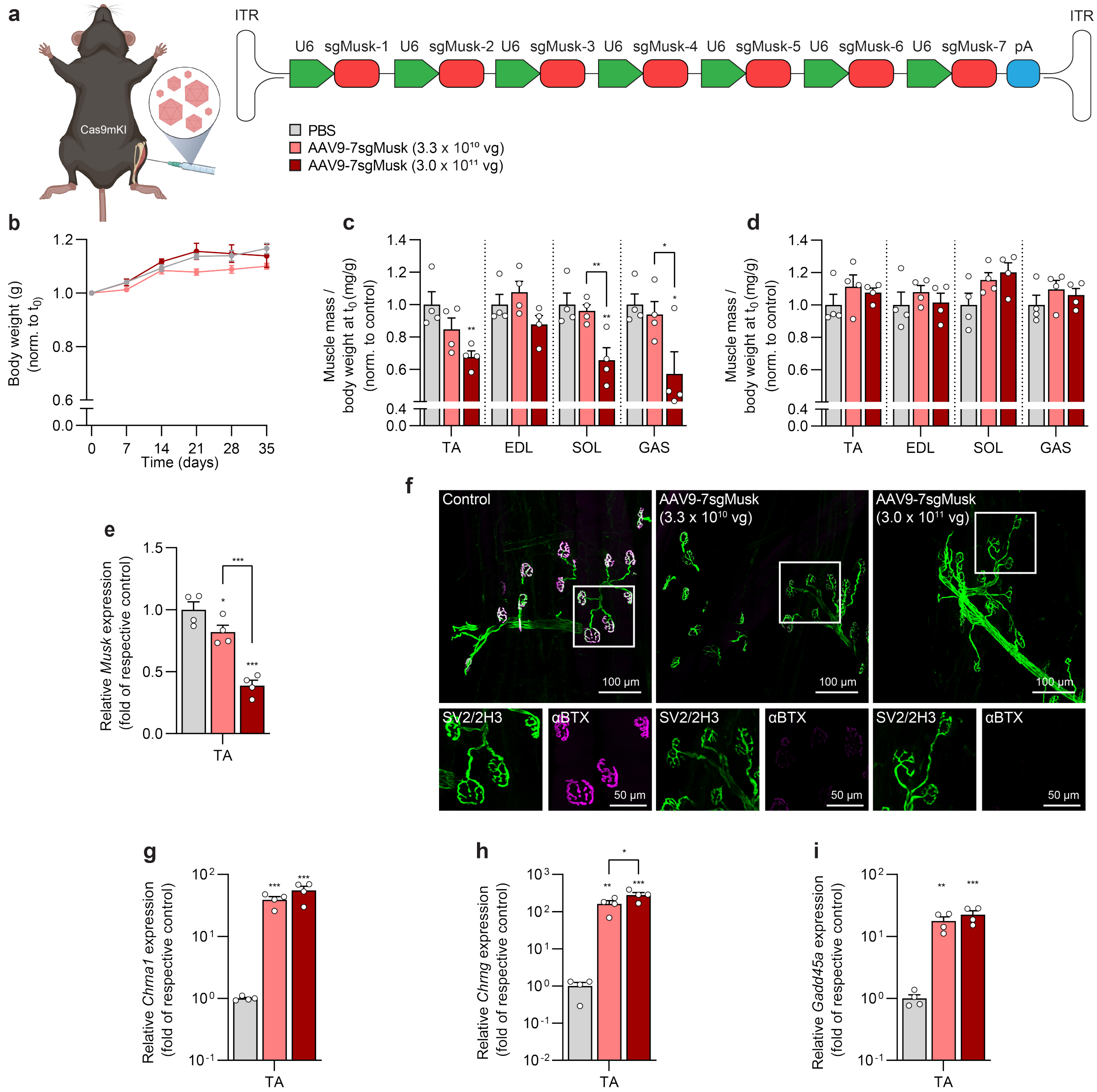

Figure S12: **Intramuscular AAV9-7sgMusk injection does not spread systemically but results in the local loss of NMJs. a** Schematic presentation of the experimental procedure using two doses of sgRNA-delivering AAV9. **b** Body weight progression of controls (grey) and AAV9-7sgMusk-injected Cas9mKI mice (red colors) at the indicated doses. Mass changes in muscles of the injected (**c**) and the contralateral leg (**d**). Muscles are *tibialis anterior* (TA), *extensor digitorum longus* (EDL), *soleus* (SOL) and *gastrocnemius* (GAS). **e** Relative mRNA expression of *Musk* in AAV9-7sgMusk-injected TA muscle of Cas9mKI mice. **f** Representative whole-mount images of *extensor digitorum longus* (EDL) muscles of controls and AAV9-7sgMusk-injected Cas9mKI mice.

Presynaptic nerve terminals are stained with a mixture of antibodies directed against synaptic vesicle glycoprotein 2A (SV2; green) and neurofilament (2H3; green). Fluorescently-labeled α-bungarotoxin (αBTX; magenta) was used to visualize postsynaptic AChRs. **g – i** Relative mRNA expression of denervation marker genes as indicated in AAV9-7sgMusk-injected TA muscles of Cas9mKI mice and of controls. Data are means ± SEM. n = 4 mice. Statistical significance is based on one-way ANOVA with Fishers LSD post-hoc test. *P < 0.05, **P < 0.01, ***P < 0.001. Experimental scheme in **a** was created with BioRender.com. Source data and precise p-values are provided as a source data file.

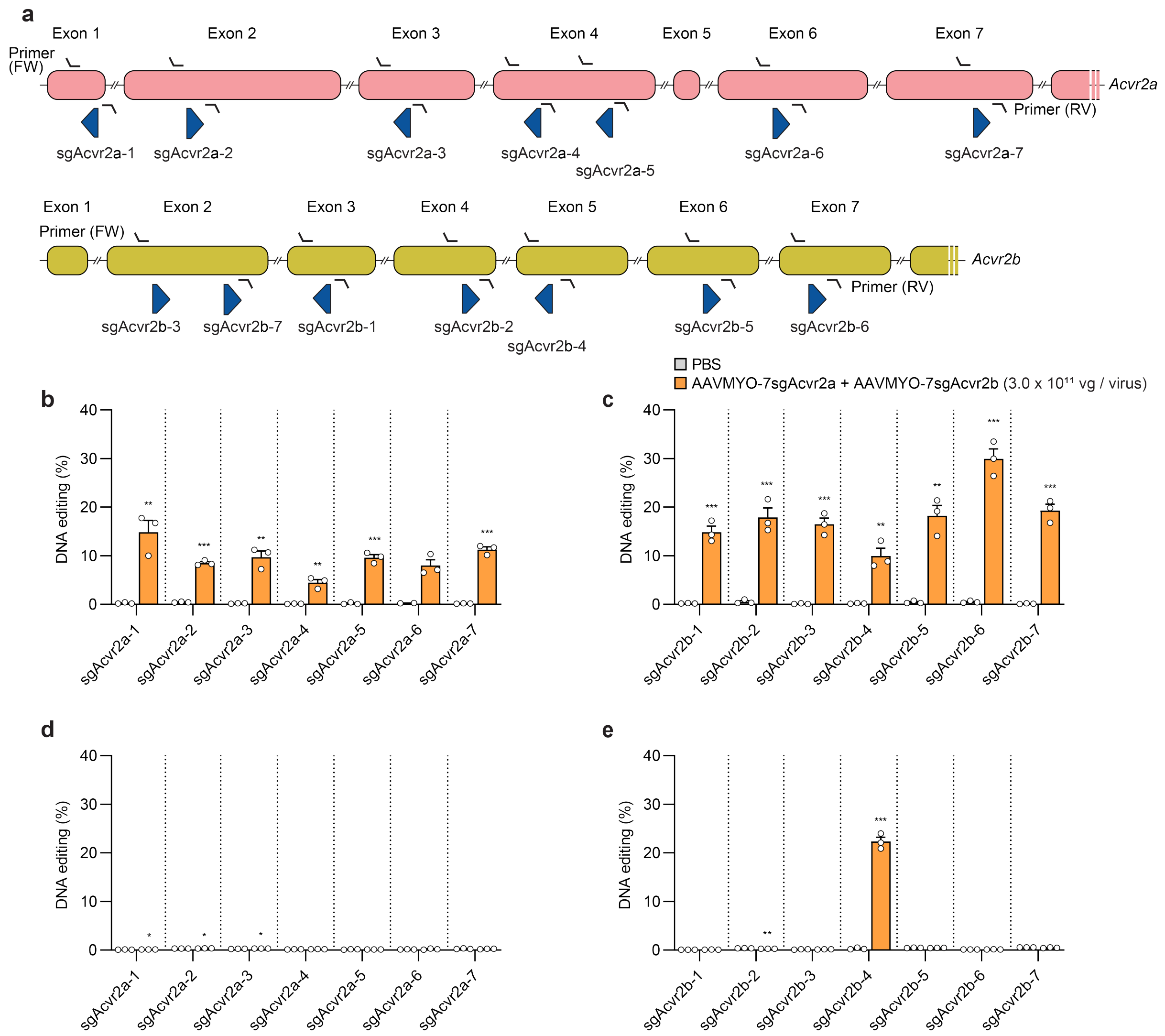

Figure S13: **On/off-target editing by individual sgAcvr2a-(1-7) and sgAcvr2b-(1-7). a** Schematic representation of amplicon design for each sgAcvr2a-(1-7) and sgAcvr2b-(1-7). Location and orientation of each sgRNA, and forward (FW) and reverse (RV) primers for each amplicon are indicated. **b, c** CRISPResso2 analysis for each sgAcvr2a (**b**) and for each sgAcvr2b (**c**) at its target site. **d, e** CRISPResso2 analysis for each sgAcvr2a (**d**) and for each sgAcvr2b (**e**) at its primary off-target site. Note that sgAcvr2b-4 has a strong editing activity at the off-target site. DNA was isolated from *tibialis anterior* (TA) muscle from PBS- (control; grey) or AAVMYO-7sgAcvr2a/b-injected Cas9mKI mice (orange). n = 3 mice. Note that there were only n = 2 for the control sample of sgAcvr2a-6 in **b**. Statistical significance is based on unpaired student's two-sided t-test. *P < 0.05, **P < 0.01, ***P < 0.001. Source data and precise p-values are provided as a source data file.

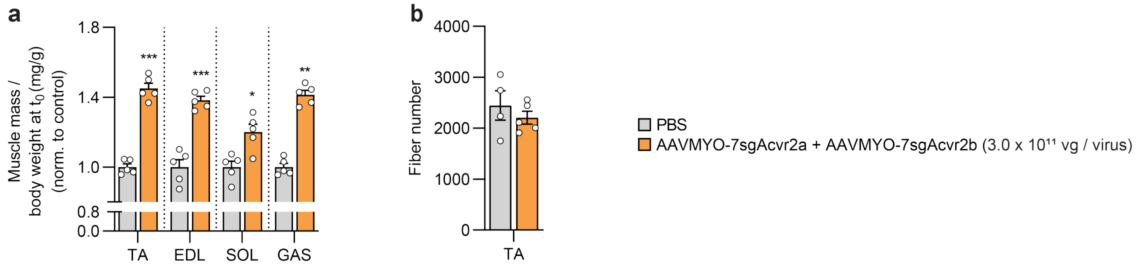

Figure S14: **AAVMYO-CRISPR/Cas9-mediated double knockout of Acvr2a/Acvr2b induces systemic hypertrophy without hyperplasia. a** Changes in mass of *tibialis anterior* (TA), *extensor digitorum longus* (EDL), *soleus* (SOL) and *gastrocnemius* (GAS) muscles of the contralateral, non-injected leg of AAVMYO-7sgAcvr2a/b- (orange) and PBS-injected mice (grey). **b** Total fiber number of TA muscles injected with PBS (grey) or AAVMYO-7sgAcvr2a/b. Data are means ± SEM. For **a**, n = 5 mice. For **b**, n = 4 (control) and 5 (AAVMYO-7sgAcvr2a/b) mice. Statistical significance is based on unpaired student's two-sided t-test. *P < 0.05, **P < 0.01, ***P < 0.001. Source data and precise p-values are provided as a source data file.

sgRNA	Predicted off-target	Off-target sequence
sgAcvr2a-1	mm3_intron_Sbf2_chr7_110364457	AAAGCAAAAGAAAAGAAAGA
sgAcvr2a-2	mm4_intron_Tcof1_chr18_60826196	TGAAAAACAACCAACCAGAC
sgAcvr2a-3	mm4_intergenic_Ostm1|Gm15199_chr10_42714315	AAAAAAAAAAGTACAATTCA
sgAcvr2a-4	mm2_intergenic_Gm9931|Gm22966_chr1_147445447	AGCAAAATGTTGTAATAGAG
sgAcvr2a-5	mm4_intergenic_Gm12381|Gm12382_chr4_38615892	GAGTAAGAAAAAATGCAGGA
sgAcvr2a-6	mm3_intron_Zpld1_chr16_55279030	TTTGTTAGAAGTGAAAACAA
sgAcvr2a-7	mm4_intron_C2cd2_chr16_97916099	AAAGAAAAGAAACACAAGTG
sgAcvr2b-1	mm4_intergenic_Glrx3|Gm25798_chr7_137915294	ACAGCAACAGAAGAAAACCT
sgAcvr2b-2	mm4_intergenic_Gm22230|Gm23558_chr12_97300204	TATCATTGGAAGCCTACCTA
sgAcvr2b-3	mm4_intron_Aldh18a1_chr19_40567915	CTGGAACACTCCAGCCAGAG
sgAcvr2b-4	mm2_intergenic_Gm24632|Lyzl1_chr18_3905757	CATGGCTTCAGGCCCACCAG
sgAcvr2b-5	mm3_intergenic_Ednra|4933431K23Rik_chr8_77740885	TCCAGTGCTGCCAAGAAACG
sgAcvr2b-6	mm4_intergenic_Gm25787|Gm23345_chr7_111902261	GTGGAAAGTTCTGTGCCACA
sgAcvr2b-7	mm4_intron_Tnxb_chr17_34675648	ACCACAGAGCTAATGAAGAA
sgMusk-1	mm4_intergenic_Gm25875|Gm23835_chr15_60213279	AAGAGATATGGAGATGACAG
sgMusk-2	mm3_intron_Pxn_chr5_115533566	CCTCCGCGCTCGGAATGGTG
sgMusk-3	mm4_intergenic_Car10|Gm11502_chr11_93604414	TTCTGTAACCTGGATAAAGG
sgMusk-4	mm4_intergenic_Gldn|1700104A03Rik_chr9_54300012	TGCAAGACAGAGCCACTGTG
sgMusk-5	mm4_exon_Alas2_chrX_150552312	AATCCATCTTAAGGCAACCA
sgMusk-6	mm2_intron_Hdac7_chr15_97812536	CAGAGCACCACAGCTCTCCA
sgMusk-7	mm4_intergenic_4930519F16Rik|Gm14847_chrX_103234460	TAGAACTATAAAGGAACCAA
sgPrkca-1	mm4_intron_Rcsd1_chr1_165682564	AGGCCATGGAGTCATCGGCC
sgPrkca-1	mm4_intergenic_Gm26993|Tle4_chr19_14416599	GGCCAAGGGCTGCATGGCGT
sgPrkca-1	mm4_intergenic_Olfr1364|Olfr1362_chr13_21584140	AAGCCCTAGAGTCCTTGGCC
sgPrkca-1	mm4_intergenic_Gm11581|Ctla4_chr1_60857766	ACGCCAGGAAGTCGTTGGCA

Table S1: **Predicted off-targets for sgRNAs**
