# Supplementary material for: Fast, multiplexable and efficient somatic gene deletions in adult mouse skeletal muscle fibers using AAV-CRISPR/Cas9
Source: Nat Commun. 2023 Sep 30;14:6116. doi: 10.1038/s41467-023-41769-7 (PMC10542775; doi:10.1038/s41467-023-41769-7)
Supplement: Supplementary file 2 — Reporting summary [file 41467_2023_41769_MOESM2_ESM.pdf]

nature portfolio

Corresponding author(s): Markus A. Rüegg

Last updated by author(s): Sep 5, 2023

Reporting Summary

Nature Portfolio wishes to improve the reproducibility of the work that we publish. This form provides structure for consistency and transparency in reporting. For further information on Nature Portfolio policies, see our Editorial Policies and the Editorial Policy Checklist.

Statistics

For all statistical analyses, confirm that the following items are present in the figure legend, table legend, main text, or Methods section.

n/a	Confirmed	
☐	☒	The exact sample size (*n*) for each experimental group/condition, given as a discrete number and unit of measurement
☐	☒	A statement on whether measurements were taken from distinct samples or whether the same sample was measured repeatedly
☐	☒	The statistical test(s) used AND whether they are one- or two-sided<br>*Only common tests should be described solely by name; describe more complex techniques in the Methods section.*
☒	☐	A description of all covariates tested
☐	☒	A description of any assumptions or corrections, such as tests of normality and adjustment for multiple comparisons
☐	☒	A full description of the statistical parameters including central tendency (e.g. means) or other basic estimates (e.g. regression coefficient) AND variation (e.g. standard deviation) or associated estimates of uncertainty (e.g. confidence intervals)
☐	☒	For null hypothesis testing, the test statistic (e.g. *F*, *t*, *r*) with confidence intervals, effect sizes, degrees of freedom and *P* value noted<br>*Give P values as exact values whenever suitable.*
☒	☐	For Bayesian analysis, information on the choice of priors and Markov chain Monte Carlo settings
☒	☐	For hierarchical and complex designs, identification of the appropriate level for tests and full reporting of outcomes
☒	☐	Estimates of effect sizes (e.g. Cohen's *d*, Pearson's *r*), indicating how they were calculated

Our web collection on statistics for biologists contains articles on many of the points above.

Software and code

Policy information about availability of computer code

Data collection	Amplicon deep sequencing FASTQ files were analyzed using the CRISPResso2 software (2.2.12) package. (Clemet et al, 2019, Nature Biotechnology). FASTQ files were aligned to the genome using STAR (2.7.9) (Dobin et al, 2013, Bioinformatics) sgRNA and respective off-targets were predcited using CRISPOR 5.01 (Jean-Paul Concordet et al, 2018, Nucleic Acids Research)
Data analysis	Data were analysed using GraphPad Prism 8.0.2. Sashimi plot was created using Integrative Genomics Viewer 2.16.1

For manuscripts utilizing custom algorithms or software that are central to the research but not yet described in published literature, software must be made available to editors and reviewers. We strongly encourage code deposition in a community repository (e.g. GitHub). See the Nature Portfolio guidelines for submitting code & software for further information.

Data

Policy information about availability of data

All manuscripts must include a data availability statement. This statement should provide the following information, where applicable:

- Accession codes, unique identifiers, or web links for publicly available datasets
- A description of any restrictions on data availability
- For clinical datasets or third party data, please ensure that the statement adheres to our policy

No datasets have been used or generated for this manuscript. Source data, full uncropped Western blots and precise p-value are provided as source data file with this paper. There is no restriction on data availability.

Research involving human participants, their data, or biological material

Policy information about studies with human participants or human data. See also policy information about sex, gender (identity/presentation), and sexual orientation and race, ethnicity and racism.

Reporting on sex and gender	No human samples were included in this research project.
Reporting on race, ethnicity, or other socially relevant groupings	No human samples were included in this research project.
Population characteristics	No human samples were included in this research project.
Recruitment	No human samples were included in this research project.
Ethics oversight	No human samples were included in this research project.

Note that full information on the approval of the study protocol must also be provided in the manuscript.

Field-specific reporting

Please select the one below that is the best fit for your research. If you are not sure, read the appropriate sections before making your selection.

[x] Life sciences [ ] Behavioural & social sciences [ ] Ecological, evolutionary & environmental sciences

For a reference copy of the document with all sections, see nature.com/documents/nr-reporting-summary-flat.pdf

Life sciences study design

All studies must disclose on these points even when the disclosure is negative.

Sample size	Sample size was chosen depending on the used technique and based on previous experience with muscle, AAV and CRISPR analysis (Castets et al, 2019, Nature Communications) (Weinmann et al, 2020, Nature Communications) (Platt et al, 2014, Cell). No power calculations were performed to choose group size. At least 3 animals/cell culture samples were used per group to perform statistics.
Data exclusions	No data was excluded from this study.
Replication	Individual replicates are shown on each graph and stated in the figure legends.
Randomization	For each experimental group, animals were randomly selected from a group of mice within the same genotype and age. Covariates such as sex or bodyweight were not controlled as AAV/CRISPR efficiencies are not influenced by them.
Blinding	As the same person was conducting all animal experiments, the investigator was not blinded for group allocation. After dissection, the investigator was blinded, as tissue was labeled by mouse number, not by experimental group. The investigator was not blinded for group allocation for experiments, for which sample order mattered (e.g. Western blot).

Reporting for specific materials, systems and methods

We require information from authors about some types of materials, experimental systems and methods used in many studies. Here, indicate whether each material, system or method listed is relevant to your study. If you are not sure if a list item applies to your research, read the appropriate section before selecting a response.

Materials & experimental systems

n/a	Involved in the study	
[ ]	[x]	Antibodies
[ ]	[x]	Eukaryotic cell lines
[x]	[ ]	Palaeontology and archaeology
[ ]	[x]	Animals and other organisms
[x]	[ ]	Clinical data
[x]	[ ]	Dual use research of concern
[x]	[ ]	Plants

Methods

n/a	Involved in the study	
[x]	[ ]	ChIP-seq
[x]	[ ]	Flow cytometry
[x]	[ ]	MRI-based neuroimaging

Antibodies

Antibodies used	Western Blot:<br>PKCa (1:1000, 2056S, Cell Signaling), FLAG (1:1000, F3165, Sigma), tdTomato (1:5000, orb182397, Biorbyt), GFP (1:1000,

	11814460001, Roche), GAPDH (1:5000, 2118, Cell Signaling), HDAC4 (1:1000, 7628S, Cell Signaling), goat anti-rabbit HRP (1:10000, 111-035-003, Jackson Immuno), goat anti-mouse HRP (1:10000, 115-035-003, Jackson Immuno), donkey anti-goat HRP (1:10000, 705-035-003, Jackson Immuno)<br><br>Immunostaining:<br>GFP (1:400, A10262, Molecular Probes), MHC1 (1:50, BA-D5, DSHB), MHC2a (1:200, SC-71, DSHB), MHC2b (1:100, BF-F3, DSHB), Laminin (1:200, L9393, Sigma), goat anti-mouse 405 (1:50, 115-475-207, Jackson Immuno), goat anti-mouse 568 (1:200, A-21124, Invitrogen), goat anti-mouse 488 (1:200, A-21042, Invitrogen), donkey anti-rabbit 647 (1:300, 711-605-152, Jackson Immuno), donkey anti-chicken 488 (1:500, 703-545-155, Jackson Immuno), Neurofilament (1:2000, 2H3, DSHB), Synaptic vesicle protein (1:400, SV2, DSHB), goat anti-mouse 555 (1:500, A-21127, Invitrogen), rabbit anti-Musk (1:5000, 194T, Prof. Markus A. Rüegg), goat anti-rabbit A405 PLus (1:150, A-48254, Invitrogen)
Validation	Western Blot:<br>PKCa (1:1000, 2056S, Cell Signaling) https://www.cellsignal.com/products/primary-antibodies/pkca-antibody/2056 , FLAG (1:1000, F3165, Sigma) https://www.sigmaaldrich.com/CH/en/product/sigma/f1804?gclid=CjwKCAiA3pugBhAwEiwAWFzwdQjkmN_4h7tkpop6tRap2jeAo_oXzOVtQtpnKR8_TUPbLhNY8ujB0BoC3GkQAvD_BwE&gclsrc=aw.ds , tdTomato (1:5000, orb182397, Biorbyt) https://www.biorbyt.com/tdtomato-antibody-orb182397.html , GFP (1:1000, 11814460001, Roche) https://www.sigmaaldrich.com/CH/en/product/roche/11814460001?gclid=CjwKCAiA3pugBhAwEiwAWFzwdUKRybE3jw_zedMk5YzJjigmdujm_86aV1HqDxL3scGINWMoUWCC7BoChGUQAvD_BwE&gclsrc=aw.ds , GAPDH (1:5000, 2118, Cell Signaling) https://www.cellsignal.com/products/primary-antibodies/gapdh-14c10-rabbit-mab/2118 , HDAC4 (1:1000, 7628S, Cell Signaling) https://www.cellsignal.com/products/primary-antibodies/hdac4-d15c3-rabbit-mab/7628 , goat anti-rabbit HRP (1:10000, 111-035-003, Jackson Immuno) https://www.jacksonimmuno.com/catalog/products/111-035-003 , goat anti-mouse HRP (1:10000, 115-035-003, Jackson Immuno) https://www.jacksonimmuno.com/catalog/products/115-035-003 , donkey anti-goat HRP (1:10000, 705-035-003, Jackson Immuno) https://www.jacksonimmuno.com/catalog/products/705-035-003<br><br>Immunostaining:<br>GFP (1:400, A10262, Molecular Probes) https://www.thermofisher.com/antibody/product/GFP-Antibody-Polyclonal/A10262 , MHC1 (1:50, BA-D5, DSHB) https://dshb.biology.uiowa.edu/BA-D5 , MHC2a (1:200, SC-71, DSHB) https://dshb.biology.uiowa.edu/SC-71 , MHC2b (1:100, BF-F3, DSHB) https://dshb.biology.uiowa.edu/BF-F3 , Laminin (1:200, L9393, Sigma) https://www.sigmaaldrich.com/CH/en/product/sigma/l9393 , goat anti-mouse 405 (1:50, 115-475-207, Jackson Immuno) https://www.jacksonimmuno.com/catalog/products/115-475-207 , goat anti-mouse 568 (1:200, A-21124, Invitrogen) https://www.thermofisher.com/antibody/product/Goat-anti-Mouse-IgG1-Cross-Adsorbed-Secondary-Antibody-Polyclonal/A-21124 , goat anti-mouse 488 (1:200, A-21042, Invitrogen) https://www.thermofisher.com/antibody/product/Goat-anti-Mouse-IgM-Heavy-chain-Cross-Adsorbed-Secondary-Antibody-Polyclonal/A-21042 , donkey anti-rabbit 647 (1:300, 711-605-152, Jackson Immuno) https://www.jacksonimmuno.com/catalog/products/711-605-152 , donkey anti-chicken 488 (1:500, 703-545-155, Jackson Immuno) https://www.jacksonimmuno.com/catalog/products/703-545-155 , Neurofilament (1:2000, 2H3, DSHB) https://dshb.biology.uiowa.edu/2H3 , Synaptic vesicle protein (1:400, SV2, DSHB) https://dshb.biology.uiowa.edu/SV2, goat anti-mouse 555 (1:500, A-21127, Invitrogen) https://www.thermofisher.com/antibody/product/Goat-anti-Mouse-IgG1-Cross-Adsorbed-Secondary-Antibody-Polyclonal/A-21127, Musk (1:5000, 194T, Prof. Markus A. Rüegg) https://doi.org/10.1016/j.expneurol.2011.04.018, Goat anti-rabbit A405 Plus (1:150, A-48254, Invitrogen) https://www.thermofisher.com/antibody/product/Goat-anti-Rabbit-IgG-H-L-Highly-Cross-Adsorbed-Secondary-Antibody-Polyclonal/A48254

Eukaryotic cell lines

Policy information about cell lines and Sex and Gender in Research

Cell line source(s)	C2C12 myoblasts/myotubes from ATCC (CRL-1772)<br>HEK293T cells from ATCC (CRL-3216)
Authentication	C2C12: Cells were authenticated by confirming their fusion into myotubes upon differentiation initiation by reducing serum (2%) in the medium. Fusion capacity and myotube morphology were observed by bright-field microscopy.<br>HEK293T: Cells were tested for yielding high rAAV titers. HEK293T cells were transfected with rAAV-production plasmid mix and rAAV yield upon rAAV isolation was quantified using qPCR targeting the rAAV genome.
Mycoplasma contamination	Mycoplasma screening was not performed.
Commonly misidentified lines<br>(See ICLAC register)	None

Animals and other research organisms

Policy information about studies involving animals; ARRIVE guidelines recommended for reporting animal research, and Sex and Gender in Research

Laboratory animals	Cas9mKI/iCas9mKI mice and WT controls were bred on a C57BL/6JRj background. For AAV administration, only mice older than 6 weeks were selected.
Wild animals	This study did not involve wild animals.
Reporting on sex	This study included male and female mice. Sex was not considered in this study. If appropriate, data was normalized to body weight for sex-normalization.

Field-collected samples	This study did not involve field-collected samples.
Ethics oversight	All procedures involving animals were performed in accordance with Swiss regulations and approved by the veterinary commission of the canton Basel Stadt.

Note that full information on the approval of the study protocol must also be provided in the manuscript.
