# Supplementary material for: Fast, multiplexable and efficient somatic gene deletions in adult mouse skeletal muscle fibers using AAV-CRISPR/Cas9
Source: Nat Commun. 2023 Sep 30;14:6116. doi: 10.1038/s41467-023-41769-7 (PMC10542775; doi:10.1038/s41467-023-41769-7)
Supplement: Supplementary file 4 — Description of Additional Supplementary Files [file 41467_2023_41769_MOESM4_ESM.pdf]

Title: Supplementary Data 1:

Description: Amplicon NGS analysis of sgPKCα-1 (Sheet 1), sgMusk(1-7) (Sheet 2) and sgAcvr2a/b-(1-7) (Sheet 3) editing at target and predicted off-target loci.

Title: Supplementary Data 2:

Description: (Sheet 1) Sequences of sgRNA and oligonucleotides used in this study. (Sheet 2) Dilution, catalog number, company and validation references of antibodies used in this study.
